# Lymphatics

**Published:** 1891-05

**Authors:** B. F. King


### LYMPHATICS.

BY B. F. KING, V. S.

The lymphatics are a system of vessels accompanying the venous system through the body, and are divided into two groups, the lymphatics and lacteals. The latter are exclusively confined to the small intestines; while the former are found in all parts of the body except the brain, eyeball, cartilage, tendons, horn and hair, and is termed the absorbent system. The history of the discovery of what is termed the absorbent system of vessels, dates from the vague allusion made by Hippocrate, Aristotle and others, to the description of the thoracic duct, in the middle of the sixteenth century, by Eustachius, and finally to the discovery of the lacteals by Asellius, in 1622, is more interesting in an anatomical, than in a physiological point of view. Our knowledge of anatomy of the absorbents dates from the discovery of the thoracic duct; and the discovery of the lacteals by Asellius dates the history of these vessels as carriers of nutritive matter from the intestinal canal to the system. In 1649, Pecquet discovered the recepticle for chyle and demonstrated that lacteals did not pass the liver, but emptied the chyle into the thoracic duct by which it was finally conveyed to the venous system.

In 1651 the history of the absorbents was more fully completed by Rudbeck, who discovered a system of vessels carrying a colorless fluid through all parts of the body, and also demonstrated their identity with the lacteals. They were afterward studied carefully by Bartholinus who gave them the name of lymphatics. The old idea which dates from the discovery by Asellius and Pecquet that the lacteals absorb all the product of digestion, was overthrown by Magendie and others, who experimented upon vascular absorption.

One of the most difficult problems in anatomy is to determine the situation and origin of the lymphatics in different parts of the system. The tenuity of the wells of these vessels which render it impossible to study them by the ordinary methods of injection. Since it has been ascertained that the injection of a solution. generally Mercury, gently diffused into the vessel of origin follows the vessels as the fluid passes along their course into the larger trunks, and thence to the tymphatic glands, the regularity with which the minute vessels carry their burden through to the larger vessels to the glands, is positive proof that the lymphatics have been penetrated, and that the appearances observed are not the result of mere infiltration.

The mode of origin of the finest vessels in the lymphatic radicles is exceedingly obscure notwithstanding the numerous investigations which have been made within the last few years, particularly by the German anatomists. In fact, lymphatics have not been actually injected and demonstrated, in all the tissues of the body. But, because we have not been able to inject them, we are not justified in assuming positively that they do not exist. For example, in the intestinal villi, according to Sappey, they have never been seen, although we have no doubt of their existence. In the elaborate observations made by Dr. Belaiff, of St. Petersburg, speaking of the origin of the lymphatics of the penis and walls of the vessels, they were made apparent by the actions of nitrate silver in solution in pure water ; and it is probable that they were very little distended for the smallest of these vessels had a diameter of about one three-hundredth of an inch.

Robins and his associates since found a curious system of vessels which inhabit the brain and spinal cord, entirely surrounding the capillary blood vessels and connected with the lymphatic trunk or reservoir described by Fohman under the pia-mater. The capilliary vessels thus float in a fluid contained in these cylindrical sheaths which exceed them in diameter about one twelve-hundredth of an inch. These investing vessels follow the blood vessels in their ramification and contain a clear fluid with bodies resembling the lymph corpuscle. When Robins first described these vessels minutely, he did not state definitely their physiological relation, but since, he has published a memoir in which he has described them as true lymphatic vessels, analagous to the lymphatic which partly surround the small vessels in fishes, reptiles, etc. In these animals the lymphatics in many parts nearly surround the blood vessels, to the walls which the edges of their proper coats are adherent, and that portion of the wall of the blood vessels which is thus enclosed, forms at the same time the walls of the lymphatics.

This disposition of the lymphatics of the brain and spinal cord, as found by Robins, would allow of free interchange by endomosis and exosmosis of the liquid portions of the blood and lymph The lymphatic glands are arranged in groups in different parts of the body. They are oval in form and of a pinkish color and vary in size from a pea to a walnut. Each is covered by a capsule which, by numerous prolongations, forms a reticular framework in the interior of the gland. As the lymphatic vessels enter the gland their external coating becomes continuous with the capsule; they then divide into numerous minute branches which, after a twisting, winding way, unite into two or more vessels which, in leaving the gland, become again enveloped in their outer coat. In the interior of the glands the vessels are surrounded by granular cells and a plexus of capillary blood vessels.

The lacteals derive their name from the milky appearance of the fluid which they convey. They are the mesenteric absorbents, and between its layers they are supported. They take their origin from the small intestines, absorb the chyle which has been produced by digestion, and after passing through two or three absorbent glands, empty their contents into the receptaculum chyli. It is now known that the fatty portions of food reduced to a very fine emulsion by the pancreatic fluid are absorbed by this system of vessels, and that these are the only principles that are taken up in large quantities. The examples that I have already shown from these scientists are enough to establish the fact. If the abdomen of a living animal be opened during full digestion, then and then only will the lacteals and thoracic duct be found distended with fatty emulsion. These vessels do not appear in the mesentery until the food has passed the orifice of the pancreatic duct. The observations of Sanders and Bouchardet remove all doubt as to the absorption of the products of the digestion, of fatty matter, by the lacteals, for these observers not only found that in dogs the proportion of fat in the chyle was increased, with an increase of fat taken in their food, but that the particular kind of fat given to the dog was recognized in the chyle. We have also seen that a certain portion escapes the lacteals and is absorbed directly by the blood vessels, and it is an important question to determine at the present day whether the lacteals, in addition to their more prominent functions, are not concerned in the absorption of drinks, the albuminoids, salines and saccharine matters.

The functional effects of this system are very important and extremely active in the constitution at large, for we are certain that the various organs of the body are continually changing wholly or partially their component parts, either for veneration or alteration, while it appears to be the office of the arteries to build up new parts and remove the waste of others—the old ones being removed by lymphatic absorption. By this wonderful power the roots of the temporaneous teeth are thus absorbed, that their crowns may more easily give way. It is in this manner that the vascular cartilages are taken up by the absorbents to make room for the bony deposits, when the animal approaches maturity. It is by the lymphatics that the dead parts are separated from the living in sloughing and ulceration, and by them that coagulated lymph and extravasated blood is removed.

The functional office of the lymphatics most important, is for the preservation of life under casualities. Long fasting is thus borne. Their capability of displacing the animal oil or marrow from the bones and the fat from the body is employed to make up the want. Hibernating animals live during their torpidity by a slow absorption of adipose matter. The absorbents seem to possess a power also of selecting the matter they take. The lacteals exclusively employ themselves in the absorption of chyle.

There is some difference of opinion among anatomists concerning the relative structure of the lymphatic glands. Some regard them as composed simply of a plexus of lymphatic vessels, held together by a delicate stroma of fibrous tissue, while others deny that there is any direct communication between the afferent and the affluent vessel, assuming that the vessels which penetrate the glands break up into small branches which open into a parenchma or gland with closed follicles, and that the fluids are collected from the gland by a second set of capillaries connected with the efferent lymphatics. It is probable that the lymphatics and lacteal vessels have no direct connection with the blood vessels except by the two openings by which they discharge their contents into the venous system, but the absorbent system shows that they not only collect fluids from the intestinal canal during digestion, but from nearly every tissue or organ in the body, and that these fluids are received into the venous circulation.

